# Novel Steroidal Components from the Underground Parts of *Ruscus aculeatus *L

**DOI:** 10.3390/molecules171214002

**Published:** 2012-11-26

**Authors:** Simona De Marino, Carmen Festa, Franco Zollo, Maria Iorizzi

**Affiliations:** 1 Dipartimento di Chimica delle Sostanze Naturali, Università degli Studi di Napoli “Federico II”, Via D. Montesano 49, I-80131 Napoli, Italy; 2 Dipartimento di Bioscienze e Territorio, Università degli Studi del Molise, Contrada Fonte Lappone, I-86090 Pesche (Isernia), Italy

**Keywords:** Ruscaceae, *Ruscus aculeatus* L., furostanol saponins, sulphated steroidal glycosides, NMR spectroscopy

## Abstract

Two new furostanol saponins **1**–**2** and three new sulphated glycosides **3a**,**b** and **4** were isolated from the underground parts of *Ruscus aculeatus *L., along with four known furostanol and one spirostanol saponins **5**–**9** and three free sterols. All of the structures have been elucidated on the basis of spectroscopic data 1D and 2D NMR experiments, MS spectra and GC analyses.

## 1. Introduction

*Ruscus aculeatus* L. (Ruscaceae family) is a small evergreen shrub and a widely distributed European plant. The hydroalcoholic extract of its rhizome is commonly used as a vascular preventive and vascular tonic in pharmaceutical preparations [1]. Previous chemical analysis of secondary metabolites have been described in *R. aculeatus* L. [2,3], *R. hypoglossum* L. [4], *R. colchicus* Y. Yeo [5] and *R. ponticus* Wor. [6]. The steroidal constituents of this herb, including spirostane, furostane and triterpene type, are the main secondary metabolites isolated from the rhizome and leaves [7].

**Figure 1:**
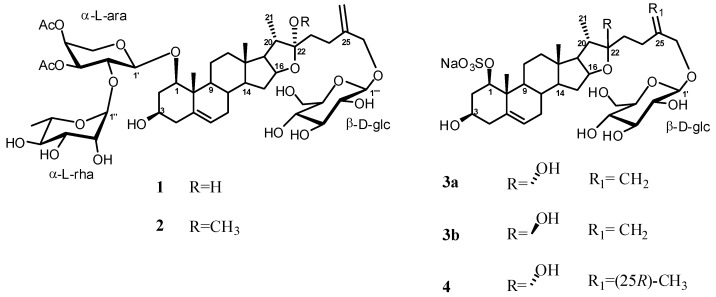
New compounds isolated from *Ruscus aculeatus* L.

**Figure 2:**
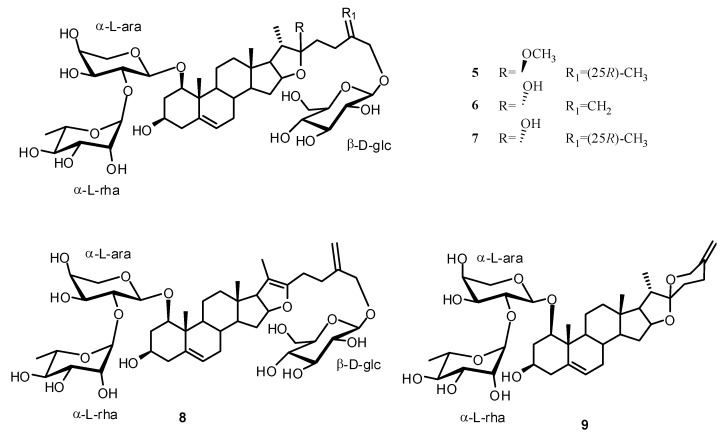
Known compounds isolated from *Ruscus aculeatus *L.

In the present work we performed a phytochemical investigation of the fresh underground parts of *Ruscus aculeatus *L. Two new furostanol saponins **1**–**2** and three new sulphated glycosides **3a**,**b** and **4** were isolated from its methanolic extract along with four known furostanol and one spirostanol glycosides **5**–**9** (Figure 1 and Figure 2). 

The composition of free sterols was also determined and campesterol, stigmasterol, sitosterol were the major components. All new components are bisdesmosidic saponins with a diglycoside moiety linked at C-1 and a glucose unit linked at C-26. The isolation of sulphated compounds in *Ruscus aculeatus* L. are previously reported only by Oulad-Ali *et al.* [8].

Their structures were determined by spectroscopic methods, including 1D and 2D NMR techniques, HRESI-MS, and chemical methods. Herein, we report the isolation and structural elucidation of the new compounds **1**–**4**.

## 2. Results and Discussion

### Structure Analysis and Characterization of Compounds **1**–**4**

Compound **1** was obtained as a white amorphous solid. The molecular formula was determined to be C_48_H_74_O_20_ from the molecular ion peak [M+Na]^+^ at *m/z* 993.4698 (calcd. for C_48_H_74_O_20_Na, 993.4671) in the positive HRESI-MS. The analysis of the ^1^H-NMR spectrum of **1**, in combination with HSQC data, showed signals for three anomeric protons δ_H_ 4.95/δ_C_ 101.7, δ_H_ 4.50/δ_C_ 100.1 and δ_H_ 4.28/δ_C_ 103.1, suggesting the presence of three sugar moieties.

In addition, the ^1^H-NMR spectrum (Table 1) showed signals for two tertiary methyl groups at δ_H_ 1.09 and 0.86 (each 3H, s) and one secondary methyl group at δ_H_ 1.02 (3H, d, 6.8 Hz), as well as one signal for a trisubstituted double bond (δ_H_ 5.55, 1H, br d, 5.6 Hz) and for an exometylene group (δ_H_ 5.09 and 4.92 each 1H, br s), three methine proton signals at δ_H_ 4.56 (1H, m, H-16), 3.38 (1H, m, H-1) and 3.33 (1H, m, H-3) indicative of secondary alcoholic functions and two methylene proton signals at δ_H_ 4.33 and 4.13 (each 1H, d, 12.5 Hz) ascribable to a primary alcoholic function. 

The ^13^C-NMR spectrum showed three secondary alcoholic functions at δ_C_ 83.9, 81.8 and 68.6, one primary alcoholic function at δ_C_ 72.4 and a hemiacetalic carbon signal at δ_C _111.7, suggesting the presence of a furostanol skeleton (Table 1). Comparison with literature data and analysis of HSQC and HMBC data revealed a furosta-5,25(27)-diene-1β,3β,22α,26-tetrol moiety. Glycosylation shifts on the aglycone were observed for C-1 (δ_C _83.9) and C-26 (δ_C_ 72.4). The C-22 α-configuration of **1** was assigned on the basis of key ROESY correlations between H-20 (δ_H_ 2.14) and the protons H_2_-23 (δ_H_ 1.84) and of the downfield shift of H-16 at δ_H_ 4.56 [9].

As concerning the sugar portion, in addition to the carbinol protons, the ^1^H-NMR spectrum (Table 2) showed signals at δ_H_ 2.03 and 2.04 (each 3H, s), ascribable to the methyl groups of two acetyl groups [δ_C_ 170.8 and 170.9 (C=O); δ_C _20.7 and 20.8 (CH_3_)], and one signal at δ_H_ 1.26 (3H, d, 6.3 Hz) indicative of a 6-deoxyhexopyranose unit (Table 2). 

The assignment of all protons and carbon chemical shifts of the three sugar units was performed by careful analysis of 2D NMR spectra, including COSY, TOCSY, HSQC and HMBC experiments, allowing the identification of one β-glucopyranosyl (Glc), one α-arabinopyranosyl (Ara) and one α-rhamnopyranosyl (Rha) units. The relatively large *J*_H1-H2_ values (7.4–8.0 Hz) indicated a β-orientation for the anomeric center of glucose and an α-orientation for that of arabinose in their pyranose form, whereas a small *J*_H1-H2_ coupling (1.2 Hz) indicated the α-configuration of the rhamnopyranosyl unit. The monosaccharides obtained from the acidic hydrolysis of **1** were identified as D-glucose, L-arabinose and L-rhamnose by GC analysis of their chiral derivatives [10].

**Figure 3:**
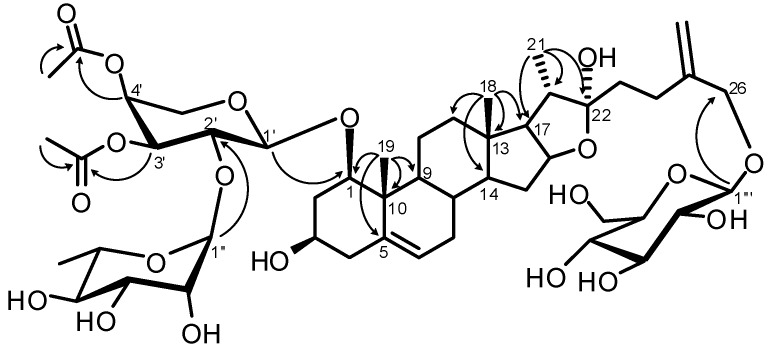
Key HMBC correlations for compound **1**.

The position of the acetyl groups at C-3′ and C-4′ of the arabinose unit was suggested by the downfield shift observed for the H-3′ (δ_H_ 5.05) and H-4′ (δ_H_ 5.30) and for the upfield shift of C-2′ (δ_C _73.5) and C-5′ (δ_C _64.5) in comparison with the data reported for the authentic sample, ruscoponticoside E (**6**), which is known compound also isolated in the present study (Table 2). These evidences were confirmed by the HMBC correlations between the proton signals at δ_H_ 5.05 (H-3′) and δ_H_ 5.30 (H-4′) with the carbonyl resonances at 170.9 ppm and 170.8 ppm (Figure 3), respectively.

The sequence and interglycosidic linkages among the three sugar units and the aglycone were revealed by HMBC experiment (Figure 3). In the HMBC spectrum, a correlation peak between H-1′′′ of Glc at δ_H_ 4.28 and C-26 at δ_C _72.4, implied that the glucose unit is attached to C-26 of the aglycone, which is a structural feature in plant furostanol saponins. The linkage of the arabinose unit to C-1 of the aglycone was ascertained by the HMBC correlation between H-1′ of arabinose (δ_H_ 4.50) and C-1 at δ_C _83.9. Furthermore the anomeric proton of rhamnose at δ_H_ 4.95 was correlated with C-2′ of arabinose at δ_C _73.5 which supported the proposed sequence of the disaccharidic chain linked at C-1 of the aglycone.

Thus the structure of compound **1** was established as 26-O-β-D-glucopyranosylfurosta-5,25(27)-diene-1β,3β,22α,26-tetrol 1-*O*-[α-L-rhamnopyranosyl-(1′′→2′)-*O*-(3′,4′-di-O-acetyl)-α-L-arabino- pyranoside].

HRESI-MS of compound **2** showed a pseudomolecular ion peak at *m/z* 1007.4857 [M+Na]^+^ corresponding to the molecular formula C_49_H_76_O_20_, which differs from that of compound **1** only in the gain of 14 u.m.a. ^1^H-NMR [δ_H_ 3.16 (3H, s)] and ^13^C-NMR [δ_C_ 113.3 (C-22) and 47.5 (-OCH_3_)] data suggested compound **2** to be a 22-methoxyfurostanol saponin (Table 1).

A HMBC experiment confirmed this hypothesis and showed a correlation between the methoxy group at δ_H_ 3.16 and C-22 (δ_C_ 113.3) of the aglycone. The ROESY experiment allowed us to assign the stereochemistry of the ketal carbon C-22. Clear correlations were observed between the methoxy group at δ_H_ 3.16 and the H-16 at δ_H_ 4.38 and between H-20 (δ_H_ 2.22) and H-23a (δ_H_ 1.90)/H-23b (δ_H_ 1.84) indicating an α-orientation of the methoxy group [3].

Analysis of COSY, HSQC and HMBC experiments revealed that **2** possessed sugar moieties identical to those of **1** (Table 2). Acidic hydrolysis of **2** afforded D-glucose, L-arabinose and L-rhamnose which were confirmed by GC analysis. Thus saponin **2** was elucidated as 26-*O*-β-D-glucopyranosyl-22α-methoxy-furosta-5,25(27)diene-1β,3β,26-triol 1-*O*-[α-L-rhamnopyranosyl-(1′′→2′)-*O*-(3′,4′-di-*O*-acetyl)-α-L-arabinopyranoside]. 

Although we have used mild extraction conditions (room temperature) we cannot exclude the possibility that compound **2** is an artifact due to reaction of compound **1** with the extraction solvent (MeOH).

Compound **3a** showed the molecular formula of C_33_H_51_O_13_S, deduced by a HRESI-MS measurement (*m*/*z *687.3043, [M−H]^−^). The presence of a sulphate group was indicated by a fragment ion peak at *m/z* 631 [M−NaSO_3_+H+Na]^+^ in the ESI-MS/MS spectrum recorded in a positive ion mode, corresponding to the loss of a SO_3_Na from the parent ion, and by IR bands at 1245 and 1086 cm^−1^.

**Table 1:** ^1^H- and ^13^C-NMR data (CD_3_OD, 500 and 125 MHz) data of the aglycon portions of compounds **1** and **2**.

Position	1		2	
	δ_H_^a^	δ_C_	δ_H_^a^	δ_C_
1	3.38 m	83.9	3.44 dd (11.8, 3.7)	83.7
2a	2.10 m	36.8	2.10 m	37.0
2b	1.69 m		1.69 m	
3	3.33 m	68.6	3.35 m	69.0
4	2.23 ovl	43.0	2.21 ovl	43.2
5	-	139.2	-	139.2
6	5.55 br d (5.6)	125.4	5.56 br d (5.4)	125.6
7a	1.97 ovl	32.3	1.96 ovl	32.6
7b	1.53 ovl		1.53 ovl	
8	1.54 ovl	33.6	1.55 ovl	33.8
9	1.25 ovl	50.9	1.25 ovl	51.1
10	-	43.5	-	43.4
11a	2.55 m	24.7	2.56 m	25.0
11b	1.49 m		1.48 m	
12a	1.68 ovl	40.7	1.70 ovl	41.1
12b	1.21 m		1.21 m	
13	-	41.6	-	41.4
14	1.13 m	57.2	1.13 m	57.5
15a	1.98 ovl	32.5	1.97 ovl	32.6
15b	1.29 m		1.26 ovl	
16	4.56 m	81.8	4.38 m	82.4
17	1.77 m	63.7	1.73 ovl	65.0
18	0.86 s	16.7	0.86 s	17.1
19	1.09 s	14.9	1.12 s	15.1
20	2.14 m	40.4	2.22 ovl	41.0
21	1.02 d (6.8)	15.4	1.04 d (6.8)	15.9
22	-	111.7	-	113.3
23a	1.84 m	37.4	1.90 m	32.1
23b			1.84 m	
24	2.29 ovl	28.3	2.18 ovl	28.5
25	-	147.4	-	147.1
26a	4.33 d (12.5)	72.4	4.34 d (12.6)	72.4
26b	4.13 d (12.5)		4.13 d (12.6)	
27a	5.09 br s	112.1	5.08 br s	112.2
27b	4.92 br s		4.93 br s	
-OCH_3_			3.16 s	47.5

Ovl: overlapped signals; ^a^ Coupling constants are in parentheses and given in Hertz. ^1^H- and ^13^C-NMR assignments aided by COSY, HSQC and HMBC experiments.

**Table 2:** ^1^H- and ^13^C-NMR data (CD_3_OD, 500 and 125 MHz) data of the sugar portions of compounds **1** and **6**.

Position	1 ^a^		6 ^b^	
	δ_H_^c^	δ_C_	δ_H_^c^	δ_C_
**α-L-Ara**				
1′	4.50 d (7.4)	100.1	4.26 d (7.1)	100.7
2′	3.85 ovl	73.5	3.70 ovl	75.4
3′	5.05 dd (9.7, 3.1)	75.7	3.64 dd (9.5, 3.2)	75.7
4′	5.30 br s	70.6	3.74 ovl	70.5
5′	3.89 ovl3.73 ovl	64.5	3.85 dd (12.1, 2.0)3.48 dd (12.1, 3.0)	67.2
3′-COCH_3_	2.04 s	20.8		
3′-COCH_3_		170.9		
4′-COCH_3_	2.03 s	20.7		
4′-COCH_3_		170.8		
**α-L-Rha**				
1′′	4.95 br d (1.2)	101.7	5.29 br d (1.2)	101.3
2′′	3.72 ovl	72.1	3.88 ovl	72.0
3′′	3.62 dd (9.6, 3.3)	71.8	3.69 ovl	71.8
4′′	3.39 t (9.6)	73.8	3.40 t (9.7)	73.8
5′′	4.08 m	69.8	4.08 m	69.5
6′′	1.26 d (6.3)	18.3	1.26 d (6.2)	18.0
**β-D-Glc (C26)**				
1′′′	4.28 d (7.7)	103.1	4.28 d (7.6)	103.0
2′′′	3.22 t (8.3)	74.9	3.21 t (8.4)	74.9
3′′′	3.35 ovl	77.9	3.35 ovl	77.9
4′′′	3.27 ovl	71.5	3.28 ovl	71.4
5′′′	3.25 ovl	77.7	3.26 ovl	77.6
6′′′	3.87 ovl3.66 dd (12.1, 4.5)	62.6	3.87 ovl3.67 dd (12.0, 4.5)	62.6

^a^ The chemical shift values of the sugar portion of **2** are identical to those reported for **1**; ^b^ Data reported for the authentic sample, ruscoponticoside E (**6**), also isolated in the present study. Ovl: overlapped signals; ^c^ Coupling constants are in parentheses and given in Hertz. ^1^H and ^13^C assignments aided by COSY, TOCSY, HSQC and HMBC experiments.

Preliminary ^1^H-NMR analysis of **3a** (Table 3) indicated the steroid glycoside nature of the compound. The ^1^H-NMR spectrum showed three methyl signals: two tertiary (δ_H_ 0.86 and 1.10) and one secondary (δ_H_ 1.04), and one anomeric proton signal at δ_H_ 4.28. Its ^13^C-NMR spectrum exhibited 33 carbon signals, with 27 being attributable to the aglycone and six attributable to the monosaccharide unit. The ^13^C-NMR spectrum further showed three secondary alcoholic functions at δ_C_ 85.5, 81.8 and 68.6, one primary alcoholic function at δ_C _72.6 and a hemiacetalic carbon signal at δ_C_ 111.1 indicating a furostane nature for the steroidal aglycone of **3a**.

Combined analysis of COSY and TOCSY experiments allowed the detection of five spin systems, four belonging to the aglycone moiety and one attributable to the monosaccharide.

**Table 3:** ^1^H- and ^13^C-NMR (CD_3_OD, 500 and 125 MHz) data of compounds **3a**, **3b** and **4**.

Position	3a		3b		4	
	δ_H_^a^	δ_C_	δ_H_^a^	δ_C_	δ_H_^a^	δ_C_
1	4.03 dd (11.9, 3.8)	85.5	4.02 dd (11.9, 3.8)	85.4	4.01 dd (11.8, 4.0)	85.6
2a 2b	2.55 m 1.69 m	38.8	2.56 m 1.69 m	38.7	2.55 m 1.67 m	38.8
3	3.43 dddd (12.0, 12.0, 6.4, 6.4)	68.6	3.41 dddd (12.0, 12.0, 6.4, 6.4)	68.6	3.45 dddd (12.1, 12.1, 6.5, 6.5)	68.7
4	2.21 ovl	43.2	2.23 ovl	43.2	2.21 m (2H)	43.0
5	-	138.7	-	139.0	-	138.8
6	5.61 br d (5.2)	126.5	5.60 br d (5.2)	126.4	5.60 br d (5.4)	126.5
7a 7b	1.98 ovl 1.55 ovl	32.8	1.99 ovl 1.57 ovl	33.0	1.96 m 1.54 ovl	32.6
8	1.55 ovl	33.8	1.56 ovl	33.8	1.54 ovl	33.8
9	1.37 ovl	50.6	1.37 ovl	50.7	1.35 ovl	50.7
10	-	43.7	-	43.6	-	43.7
11a 11b	2.36 br d (12.6) 1.52 ovl	24.0	2.35 br d (12.6) 1.52 ovl	24.0	2.36 br d (12.4) 1.48 ovl	24.0
12a 12b	1.71 ovl 1.26 m	40.8	1.70 ovl 1.28 m	40.9	1.70 ovl 1.24 ovl	40.9
13	-	41.2	-	41.4	-	41.4
14	1.17 m	57.4	1.17 m	57.5	1.18 m	57.5
15a 15b	1.99 ovl 1.32 ovl	32.7	2.00 ovl 1.33 ovl	32.6	1.95 ovl, 1.26 ovl	32.7
16	4.57 m	81.8	4.38 m	82.2	4.57 q (5.6)	82.0
17	1.78 ovl	64.0	1.74 ovl	64.1	1.71 ovl	64.1
18	0.86 s	16.8	0.87 s	16.9	0.83 s	16.9
19	1.10 s	14.5	1.10 s	14.5	1.10 s	14.6
20	2.18 ovl	40.8	2.19 ovl	40.9	2.16 ovl	41.1
21	1.04 d (6.7)	15.8	1.01 d (6.7)	16.0	0.99 d (7.1)	16.1
22	-	111.1	-	113.7	-	111.3
23a 23b	1.90 ovl 1.86 ovl	37.5	1.91 ovl 1.85 ovl	37.3	1.61 ovl	31.4
24a 24b	2.22 ovl 2.15 ovl	28.5	2.21 ovl 2.15 ovl	28.4	1.57 ovl 1.12 m	28.6
25	-	147.0	-	147.3	1.72 m	34.8
26a 26b	4.34 d (12.5) 4.11 d (12.5)	72.6	4.33 d (12.4) 4.12 d (12.4)	72.4	3.74 dd (9.4, 6.5) 3.39 ovl	75.6
27a 27b	5.09 br s 4.93 br s	111.9	5.10 br s 4.94 br s	111.9	0.95 d (6.6)	17.1
Glucose						
1′	4.28 d (7.8)	103.0	4.27 d (7.8)	103.1	4.24 d (7.7)	104.2
2′	3.22 t (8.5)	74.9	3.20 t (8.5)	75.0	3.19 t (8.4)	74.8
3′	3.36 ovl	77.8	3.34 ovl	77.8	3.35 ovl	77.9
4′	3.28 ovl	71.5	3.28 ovl	71.4	3.27 ovl	71.5
5′	3.27 ovl	77.7	3.26 ovl	77.8	3.26 ovl	77.7
6′	3.86 d (11.7) 3.66 dd (11.7, 5.2)	62.6	3.88 d (11.7) 3.65 dd (11.7, 5.2)	62.6	3.87 d (11.9) 3.68 dd (11.9, 5.1)	62.7

Ovl: overlapped signals; ^a^ Coupling constants are in parentheses and given in Hertz. ^1^H- and ^13^C- assignments aided by COSY, TOCSY, HSQC and HMBC experiments.

The location of the sulphate group at position-1 was inferred by the downfield shift of the corresponding nuclei (H-1, δ_H_ 4.03 and C-1, δ_C_ 85.5) [7]. The relative stereochemistry at C-1 and C-3 was evaluated by an accurate coupling constants analysis and by ROESY experiments. In particular, H-1 appeared as a double doublet (11.9 and 3.8 Hz), whereas H-3 appeared as a dddd with two large (ax-ax) and two small (ax-eq) coupling constants. These data pointed to the axial position of both H-1 and H-3 also confirmed by ROESY correlation of H-1 with H-3. The NMR data of side chain from C-22 to C-26, was almost superimposable to the data observed in compound **1** indicating the presence of an exomethylene function 25(27). The C-22 configuration of **3a** was assigned as α-configuration and was derived by the ROESY experiment that showed key correlations between H-20 (δ_H_ 2.18) and the protons H-23a (δ_H_ 1.90)/H-23b (δ_H_ 1.86) [3] and on the basis of the downfield shift of H-16 at δ_H_ 4.57 [9].

The linkage of the sugar to the C-26 hydroxyl group was shown by the HMBC correlation between the anomeric carbon at δ_C_ 103.0 and the two protons at C-26 (δ_H_ 4.34 and 4.11). Acidic hydrolysis of **3a** afforded D-glucose, which was confirmed by GC analysis. Thus compound **3a** was established as 26-O-β-D-glucopyranosyl-furosta-5,25(27)diene-1β,3β,22α,26-tetrol 1-O-sulphate.

The spectral data of glycoside **3b** indicated its isomeric relationship with sulphated glycoside **3a**. In fact, **3b **has the same molecular formula determined by HRESI-MS (See Experimental), and ^1^H- and ^13^C-NMR spectra (Table 3) almost identical to those of **3a**, differing only in the resonances of the carbon atom C-22 (see Table 3). This, in agreement with previous findings [11], indicated that **3b **had the opposite configuration at the hemiacetal carbon 22 (22β−ΟΗ) also supported by the H-16 resonance at δ_H_ 4.38.

COSY, HSQC and HMBC experiments showed that **3b** was substituted at its C-1 position by a sulphate group and at C-26 by a β-D-glucopyranosyl moiety, thus compound **3b** was defined as: 26-*O*-β-D-glucopyranosyl-furosta-5,25(27)diene-1β,3β,22β,26-tetrol 1-*O*-sulphate.

The HRESI-MS spectrum of **4** exhibited a pseudomolecular ion peak at *m/z* 689.3190 [M−H]^−^ (calcd. for C_33_H_53_O_13_S, 689.3207) indicating the molecular formula C_33_H_53_NaO_13_S in accordance with ^13^C NMR data. The ^1^H- and ^13^C-NMR data of the aglycone portion of compound **4**, in comparison to those of aglycone of **3a**, clearly suggested that **4** differs from **3** by the replacement of the exomethylene group with a secondary methyl group at C-27 (δ_H_ 0.95, δ_C _17.1) (Table 3). The absolute configuration of C-25 was deduced to be *R* based on the difference of chemical shifts (Δ_ab_ = δ_A_ − δ_B_) of the geminal protons H_2_-26 (Δ_ab_ = 0.35 ppm). It has been described that Δ_ab_ is usually ≥0.57 ppm in 25*S* compounds and ≤0.48 ppm in 25*R* compounds [12].

The presence of the sulphate group was confirmed after solvolysis in a dioxane-pyridine mixture that afforded a less polar desulphated derivative **4a**, which gave a pseudomolecular ion at *m/z* 615 [M+Na]^+^. The analysis of NMR spectra showed a high field shift of H-1 at δ_H_ 3.34 (*vs*. δ_H_ 4.01) and C-1 at δ_C_ 78.6 (*vs.* δ_C_ 85.6), confirming the location of the sulphate at C-1. A moderate upfield shift was observed also for the CH_3_-19 at δ_H_ 1.05 (*vs*. δ_H_ 1.10 in the natural compound). The solvolysis reaction led to the loss of a H_2_O molecule as determined by ESI-MS data and by appearance in the ^1^H-NMR spectrum of one allylic methyl group at δ_H_ 1.60 assigned to C-21.

The NMR data (COSY, TOCSY, HSQC, HMBC) for the sugar portion, were superimposable with those of compound **3a** and **3b** also confirmed by acidic hydrolysis and GC sugar analysis. Thus compound **4** was elucidated as (25*R*),26-*O*-β-D-glucopyranosyl-furost-5-ene-1β,3β,22α,26-tetrol 1-*O*-sulphate.

In previous studies on the crude extracts from the rhizome of *Ruscus aculeatus *L., Oulad-Ali *et al.* [7] reported the isolation of a compound constitutionally identical to compound **4**. The stereochemistry at C-22 was left unassigned. Comparison between the ^13^C-NMR data of the two compounds evidenced some small but not insignificant differences, pointing to a stereoisomeric relationship.

Five known compounds were additionally isolated, namely ceparoside A (**5**) [13]; ruscoponticoside E (**6**) [14]; ceparoside B (**7**) [13]; 26-*O*-β-D-glucopyranosyl-furosta-5,20(22), 25(27)-triene-1β,3β,26-triol 1-*O*-[α-L-rhamnopyranosyl-(1→2)-*O*-α-L-arabinopyranoside] (**8**) [15]; and spirosta-5,25(27)-diene-1β,3β-diol 1-*O*-[α-L-rhamnopyranosyl-(1→2)-*O*-α-L-arabinopyranoside] (**9**) [15].

Besides saponins and furostanol glycosides, the hexane extract of the rhizome contains also several minor sterols (campesterol, stigmasterol and sitosterol). The identification has been performed by means of MS spectra and NMR data and comparison with literature data. A previous study on sterol composition of *Ruscus aculeatus* L. was reported by Dunouau *et al.* [7].

## 3. Experimental

### 3.1. General

High-resolution ESI mass spectrometry (HRESI-MS) was recorded on a Micromass QTOF spectrometer and electrospray ionization mass spectrometry (ESI-MS) experiments were performed on an Applied Biosystem API 2000 triple-quadrupole mass spectrometer. Optical rotations were determined on a Jasko P-2000 polarimeter. NMR spectra were obtained on a Varian Inova 500 NMR spectrometer (^1^H at 500 MHz and ^13^C at 125 MHz) equipped with a Sun hardware, δ (ppm), *J* in Hz, using solvent signal for calibration (^13^CD_3_OD at δ_C_ 49.0 and residual CD_2_HOD at *δ*_H_ = 3.31). The Heteronuclear Single-Quantum Coherence (HSQC) spectra were optimized for an average ^1^*J*_CH_ of 140 Hz; the gradient-enhanced Heteronuclear Multiple Bond Correlation (HMBC) experiment were optimized for a ^3^*J*_CH_ of 8 Hz. 

HPLC was performed using a Waters 510 pump equipped with a Rheodyne 7125 injector and a Waters 401 differential refractometer as detector, using a Nucleodur 100-5 C_18_ column (5 µm, 4.6 mm i.d. × 250 mm); flow rate was 1 mL min^−1^. Droplet counter-current chromatography (DCCC) was performed on a DCC-A apparatus (Tokyo Rikakikai Co., Tokyo, Japan) equipped with 250 glass-columns. 

The GC/MS analysis was carried out with an Agilent Technologies 6890N Network gas chromatograph coupled to an Agilent Technologies 5973 Network quadrupole mass selective spectrometer and provided with a split/splitless injection port. Helium was used as carrier gas at a linear velocity of 40 cm/s. Separation of compounds was performed on a HP-5 MS capillary column (30 m × 0.25 mm, 0.25 µm film thickness, Agilent USA). GC oven temperature was kept constant at 180 °C. The injector temperature was 230 °C. The temperature of the ion source and the transfer line was 250 and 280 °C, respectively. Mass spectra were taken at 70 eV and the mass range was from 40 to 350 amu.

### 3.2. Plant Material

Selected samples of wild growing plants *Ruscus aculeatus *L. (Ruscaceae) were collected in May of 2009 in the mountain area of the Tuscany region in Italy. Plants were identified at the Dipartimento di Bioscienze e Territorio, (University of Molise) and a voucher specimen is deposited under No. PGT-58-09 in the Herbarium of University of Molise (Pesche, Isernia). Rhizomes were kept frozen at −20 °C until analyzed.

### 3.3. Compound Isolation

Underground fresh parts (243 g) were semi-thawed, cut and extracted with MeOH (3 × 700 mL) at room temperature. The combined extracts (56 g) were concentrated and subjected to a modified Kupchan’s [16] partitioning procedure as follows. The MeOH extract was dissolved in 10% aqueous methanol and partitioned against *n*-hexane to furnish a *n*-hexane extract (483.8 mg). The water content (% v/v) of the MeOH extract was adjusted to 40% and partitioned against CHCl_3_, to furnish a CHCl_3_ extract (3.74 g). The aqueous phase was concentrated to remove MeOH and then extracted with *n*-BuOH yielding 9.0 g of glassy material. 

The CHCl_3_ extract (1.8 g) was fractionated by DCCC using CHCl_3_/MeOH/H_2_O (7:13:8) in the ascending mode (the lower phase was the stationary phase), flow rate 8 ml/min; 4 ml fractions were collected. Fractions were monitored by TLC on SiO_2_ with CHCl_3_/MeOH/H_2_O (80:18:2) as eluent and combined on the basis of their similar TLC retention factors. Three major fractions were obtained and then separated by HPLC on a Nucleodur 100-5 C18 column (5 µm, 4.6 mm i.d × 250 mm): fraction 1 was purified with MeOH/H_2_O (65:35) as eluent, to afford 6.0 mg of known compound **5**; fraction 2 was purified with MeOH/H_2_O (7:3) to give 1.9 mg of compound **1**, 1.5 mg of compound **2**. Fraction 3 yielded known compound **9** (35.3 mg).

The *n*-BuOH extract (2.0 g) was submitted to DCCC with *n*-BuOH/Me_2_CO/H_2_O (3:1:5) in the descending mode (the upper phase was the stationary phase). The obtained fractions were monitored by TLC on Silica gel plates with *n*-BuOH/OHAc/H_2_O (12:3:5) and CHCl_3_/MeOH/H_2_O (80:18:2) as eluents. Two fractions A and B were obtained and purified by HPLC on a Nucleodur 100-5 C18 column (5 µm, 4.6 mm i.d × 250 mm). 

Fraction A (195 mg) was separated with MeOH/H_2_O (48:52) as eluent (flow rate 1 mL/min) affording 2 mg of compound **3a**, 1.9 mg of compound 3**b** and 2.6 mg of compound **4**.

Fraction B (541 mg) was purified by HPLC with MeOH/H_2_O (48:52) as eluent and contained known compounds **6** (28.8 mg), **7** (6.2 mg) and **8** (2.7 mg).

*Compound* **1**: Amorphous solid. [α]^25^_*D*_ −29.7 (*c* 0.05, MeOH); HRESI-MS *m/z* 993.4698 [M+Na]^+^ (calcd. for C_48_H_74_O_20_Na, 993.4671). The ^1^H- and ^13^C-NMR spectral data are listed in Table 1, Table 2.

*Compound* **2**: Amorphous solid. [α]^25^_*D*_ −7.3 (*c* 0.15, MeOH); HRESI-MS *m/z* 1007.4857 [M+Na]^+^ (calcd. for C_49_H_76_O_20_Na, 1007.4828). The ^1^H- and ^13^C-NMR spectral data are listed in Table 1, Table 2.

*Compound ***3a**: Amorphous solid. [α]^25^_*D*_ −70.5 (*c* 0.2, MeOH); HRESI-MS *m/z* 687.3043 [M−H]^−^ (calcd. for C_33_H_51_O_13_S, 687.3050); ESI-MS (+ve ion) *m/z* 733 [M+Na]^+^. ESI-MS/MS (+ve ion) m/z 631 [M-NaSO_3_+H+Na]^+^. IR νmax (KBr disc)/cm^−1^ 1245, 1086. The ^1^H- and ^13^C-NMR spectral data are listed in Table 3.

*Compound ***3b**: Amorphous solid. [α]^25^_*D*_ −75.3 (*c* 0.19, MeOH); HRESI-MS *m/z* 687.3030 [M−H]^−^ (calcd. for C_33_H_51_O_13_S, 687.3050); ESI-MS (+ve ion) *m/z* 733 [M+Na]^+^. The ^1^H- and ^13^C-NMR spectral data are listed in Table 3.

*Compound* **4**: Amorphous solid. [α]^25^_*D*_ −29.2 (*c* 0.26, MeOH); HRESI-MS *m/z* 689.3190 [M−H]^−^ (calcd. for C_33_H_53_O_13_S, 689.3207). The ^1^H- and ^13^C-NMR spectral data are listed in Table 3.

*Compound* **5**: Amorphous solid. [α]^25^_*D*_ −28.0 (*c* 0.60, MeOH); HRESI-MS *m/z* 925.4782 [M+Na]^+^ (calcd. for C_45_H_74_O_18_Na, 925.4773). The ^1^H- and ^13^C-NMR spectral data are consistent with the published data [12].

*Compound* **6**: Amorphous solid. [α]^25^_*D*_ −30.0 (*c* 0.93, MeOH); HRESI-MS *m/z* 909.4465 [M+Na]^+^ (calcd. for C_44_H_70_O_18_Na, 909.4460). The ^1^H- and ^13^C-NMR spectral data are consistent with the published data [13].

*Compound* **7**: Amorphous solid. [α]^25^_*D*_ −29.4 (*c *0.07, MeOH); HR-ESI-MS *m/z* 911.4623 [M+Na]^+^ (calcd. for C_44_H_72_O_18_Na, 911.4616). The ^1^H- and ^13^C-NMR spectral data are consistent with the published data [12].

*Compound ***8**: Amorphous solid. [α]^25^_*D*_ −4.63 (*c *0.08, MeOH); HRESI-MS *m/z* 891.4361 [M+Na]^+^ (calcd. for C_44_H_68_O_17_Na, 891.4354). The ^1^H- and ^13^C-NMR spectral data are consistent with the published data [14].

*Compound* **9**: Amorphous solid. [α]^25^_*D*_ −64.0 (*c *0.69, MeOH); HRESI-MS *m/z* 729.3832 [M+Na]^+^ (calcd. for C_38_H_58_O_12_Na, 729.3826). The ^1^H- and ^13^C-NMR spectral data are consistent with the published data [14].

### 3.4. Solvolysis of Compound **4** Giving **4a**

A solution of compound **4** (2.6 mg, 0.0036 mmol) in pyridine (0.5 mL) and dioxane (0.5 mL) was heated at 150 °C for 2 h in a stoppered reaction vial. After the solution was cooled, the mixture was evaporated to dryness and then purified by HPLC on a Nucleodur 100-5 C18 column (5 µm, 4.6 mm i.d. × 250 mm) with MeOH/H_2_O 8:2, to give 1.7 mg of desulphated compound **4a**. Compound **4a**: [α]^25^_D_ −7.8 (*c* 0.17, MeOH); ESI-MS: 615 [M+Na]^+^; selected ^1^H-NMR (CD_3_OD, 500 MHz) data for compound **4a**: 5.55 (1H, br d, 5.4 Hz, H-6), 4.72 (1H, m, H-16), 3.70 (1H, dd, 9.4, 6.5 Hz, H-26a), 3.39 (1H, ovl, H-26b), 3.39 (1H, ovl, H-3), 3.34 (1H, ovl, H-1), 1.60 (3H, s, H_3_-21), 1.05 (3H, s, H_3_-19), 0.95 (3H, d, 6.6 Hz, H_3_-27), 0.72 (3H, s, H_3_-18).

### 3.5. Methanolysis of **1**–**2**: Sugar Analysis

A solution of compounds **1**–**2** (0.5 mg) in anhydrous 2 N HCl-MeOH (0.5 mL) was heated at 80 °C in a stoppered reaction vial. After 2 h, the reaction mixture was cooled, neutralized with Ag_2_CO_3_, and centrifuged, and the supernatant was taken to dryness under N_2_. 1-(Trimethylsilyl)imidazole in pyridine was added and left at room temperature for 15 min. The derivatives were analyzed by GC-MS (HP-5MS capillary column, helium carrier, flow 10 mL min^−1 ^oven temperature 150 °C). GC-MS peaks in the sylilated saponin hydrolysate coeluted with those in silylated standards (methyl rhamnosides, methyl arabinosides and methyl glucosides). 

## 4. Conclutions

Two new furostanol saponins **1**–**2** and three new sulphated glycosides **3a**, **3b **and **4** were isolated from the underground parts of *Ruscus aculeatus *L., along with four known furostanol and one spirostanol saponins **5**–**9** and three free sterols. The new compounds add knowledge in the field of isolation and structural characterization of new metabolites from natural sources.
